# Supplementary material for: Influence of pump laser fluence on ultrafast myoglobin structural dynamics
Source: Nature. 2024 Feb 14;626(8000):905–11. doi: 10.1038/s41586-024-07032-9 (PMC10881388; doi:10.1038/s41586-024-07032-9)
Supplement: Supplementary file 1 — Supplementary Notes 1–3, Figs. 1–13 and Tables 1 and 2. [file 41586_2024_7032_MOESM1_ESM.pdf]

nature portfolio

https://doi.org/10.1038/s41586-024-07032-9

# Supplementary information

# Influence of pump laser fluence on ultrafast myoglobin structural dynamics

In the format provided by the authors and unedited

## Supplementary Information - Barends *et al.*, Influence of pump laser fluence on ultrafast dynamics in myoglobin

Supplementary Notes 1-3

Supplementary Figures (1-13)

Supplementary Tables (1-2)

## Supplementary Note 1: Methods and Analysis

### 1.1 Retrieval of triggered-state structures in time-resolved crystallography

Time-resolved crystallography relies on triggering a reaction in a ground-state crystal, through light activation or rapid mixing, or some other method. In any case, typically less than 100% of unit cells will be successfully triggered; in the case of light activation, this is because of the high optical densities of chromophore-containing crystals and in rapid mixing, reactant diffusion times are typically not negligible with respect to reaction time scales. Also in both cases, the triggering will not be uniform throughout the crystal; light activation will be more efficient on the light-exposed side of the crystal[26] whereas in rapid mixing, triggering will occur towards the outside of the crystal first, and deeper inside the crystal later (Figure S1).

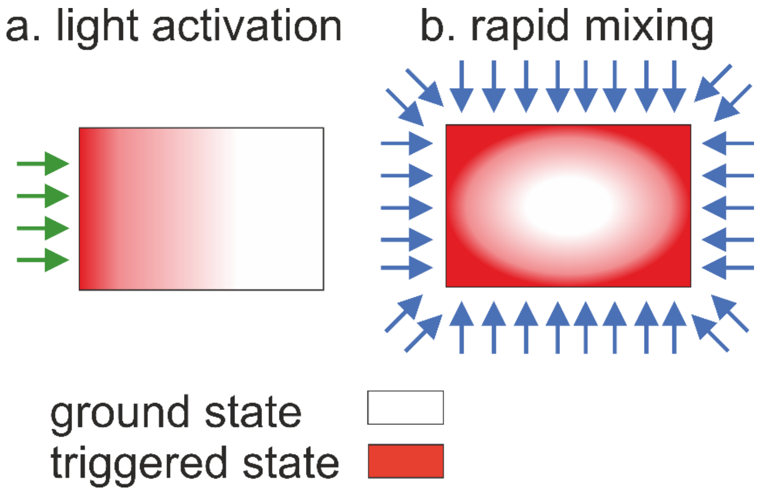


**Figure S1. Reaction initiation in crystals results in a mixture of populations. a**. light activation of a reaction in a crystal, as well as **b.** activation by rapid mixing.

Thus, the crystal will contain (at least) two types of molecules: those where triggering was successful and where the reaction under study has been initiated, and those where triggering did not occur. These will have different structures; the non-triggered molecules will remain in the ground state, whereas for those molecules in which triggering was successful, the structure will evolve over time. As the current paper uses light activation, we will use an appropriate terminology and call the ground state structure the "dark" structure, and the light-activated structure the "triggered" structure. As these structures differ, the dark- and triggered states will have different structure factors; we will call these $\vec{F}_{dark}$ and $\vec{F}_{triggered}$, respectively.

Importantly, because of this, the structure factors of the entire crystal upon triggering (which we will call $\vec{F}_{light}$ as we use light activation) will arise from a combination of $\vec{F}_{dark}$ and $\vec{F}_{triggered}$; it is usually assumed that $\vec{F}_{dark}$ and $\vec{F}_{triggered}$ sum as complex structure factors, as shown in Figure S2a. In this figure, it is assumed that 40% of the crystal has been successfully triggered and thus 60% remains in the dark state, i.e., there is 40% "occupancy" of the triggered state. $\vec{F}_{dark}$ and $\vec{F}_{triggered}$ are scaled by these factors 0.6 and 0.4 (Figure S2b) and summed in the complex plane, resulting in $\vec{F}_{light}$ (Figure S2c), of which the amplitude $|\vec{F}_{light}|$ is observed as the square root of the intensity, $I_{light}$. (For completeness, it should be mentioned that, because the triggering is inhomogeneous (see Figure S1) it is not impossible that, for certain sizes of the triggered- and non-triggered domains and certain coherence lengths of the X-rays used, the diffraction from the two domains could sum as intensities.)

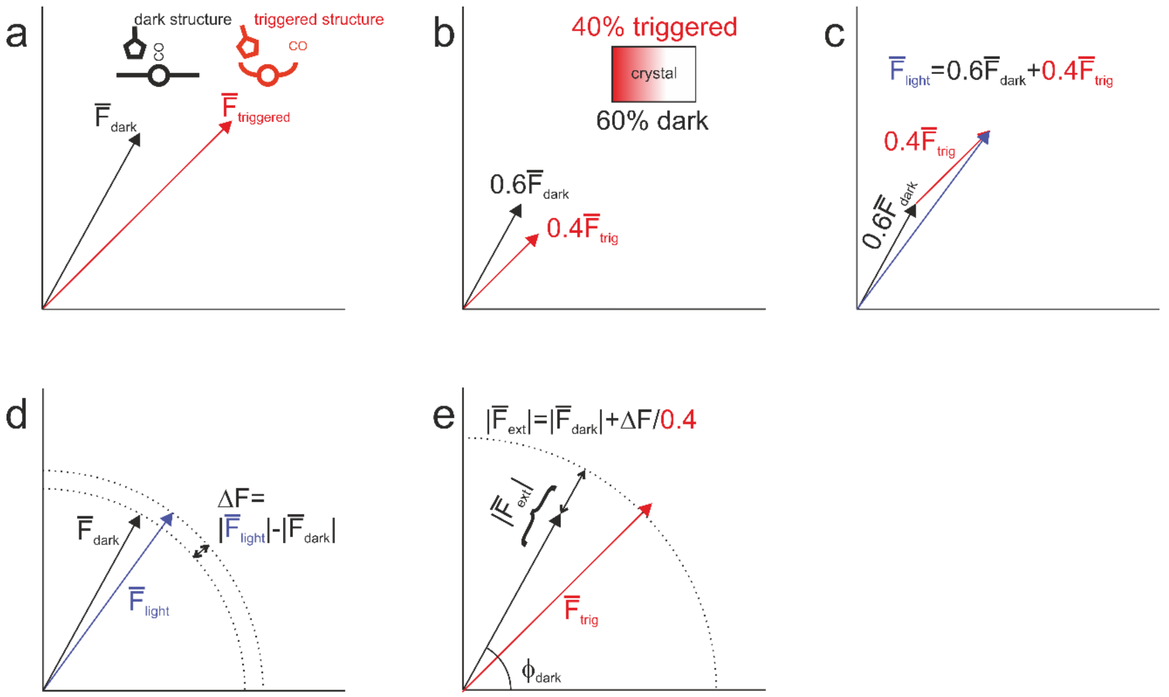


**Figure S2. Construction of the observed light-state structure factor amplitudes by complex summation of dark- and triggered state structure factors, and extrapolated structure factor calculation**. **a.** Two different structures are present in the crystal; the dark structure (black) and the triggered structure (red). Each has its own structure factors, $\vec{F}_{dark}$ and $\vec{F}_{triggered}$. **b.** These coexist in the crystal in different proportions, so their structure factors are scaled by the respective occupancies, 40% for the triggered state and 60% for the dark state in the example shown. **c.** Emanating from the crystal, however, is the complex sum of the scaled $\vec{F}_{dark}$ and $\vec{F}_{triggered}$, called $\vec{F}_{light}$ here. **d.** Of this $\vec{F}_{light}$ , the amplitude is observed (outer dashed line), which differs from the amplitude of $\vec{F}_{dark}$ (inner dashed line) observed from an untriggered crystal. In the example shown, there is only a small difference $\Delta F$ between the amplitudes of $\vec{F}_{light}$ and $\vec{F}_{dark}$, even though the amplitude of the original $\vec{F}_{triggered}$ differs much more from the amplitude of $\vec{F}_{dark}$, because of (i.) the scaling of $\vec{F}_{dark}$ and $\vec{F}_{triggered}$ by the occupancies of the respective states (panel b), and (ii.) the fact that $\vec{F}_{dark}$ and $\vec{F}_{triggered}$ have different phases with which they are added together in the complex

plane (panel c). **e.** an approximation of $|\vec{F}_{triggered}|$, the extrapolated structure factor amplitude$|\vec{F}_{ext}|$, can be obtained by scaling the amplitude difference $\Delta F$ between $\vec{F}_{light}$ and $\vec{F}_{dark}$ by the inverse of the occupancy of the triggered state to $\vec{F}_{dark}$ in a scalar manner, i.e., using the phases of $\vec{F}_{dark}$.

The problem a crystallographer is faced with is retrieving $\vec{F}_{triggered}$, but the only quantities available are the amplitudes of $\vec{F}_{dark}$ and $\vec{F}_{light}$. As shown in (Figure S2), $\vec{F}_{light}$ can be very different from the desired $\vec{F}_{triggered}$ due to the complex summation of the scaled $\vec{F}_{dark}$ and $\vec{F}_{triggered}$ : in the example shown, the large difference in amplitude between $\vec{F}_{dark}$ and $\vec{F}_{triggered}$ is largely negated by (i.) the scaling by the occupancies and (ii.) the phase difference, and the only thing that is observed upon activation in this case is a very small amplitude change $\Delta F = |\vec{F}_{light}| - |\vec{F}_{dark}|$ (Figure S2d).

Typically, the first thing one does after data collection is to calculate a "light minus dark map" from this difference, using dark-state phases. Assuming that the structural change is not too large and the dark and triggered states are isomorphous, this will result in a map that shows the change in electron density upon triggering, but the approximations result in these features being at ½ scale, and will add noise[76]. This light-minus-dark difference map will be positive where new electron density has appeared and negative where it has disappeared, and while it immediately gives information on the structural change, it is difficult to build a model of the triggered state in such a map.

Fortunately, one can approximate $\vec{F}_{triggered}$ by a process called linear extrapolation[62,63]; here, the observed light-dark amplitude difference $\Delta F$ is scaled by the inverse of the occupancy of

the triggered state, and added to $|\vec{F}_{dark}|$ as a scalar. This process is sometimes also called scalar extrapolation. The result is called an extrapolated structure factor amplitude $|\vec{F}_{extrapolated}|$.

$$|\vec{F}_{extrapolated}| = \frac{|\vec{F}_{light}| - |\vec{F}_{dark}|}{triggered\ state\ occupancy} + |\vec{F}_{dark}|$$

Calculating a map from extrapolated structure factor amplitudes using dark-state phases will approximate a map calculated using $\vec{F}_{triggered}$; it is as if the small differences resulting from partial occupancy of the triggered state have been extrapolated to 100% occupancy (Figure S2e). It is interesting to note that this is equivalent to adding an appropriately scaled light-dark difference map to the dark-state electron density map; thus, any features introduced by the extrapolation will be at ½ scale and noise will have been added in the process. Nevertheless, refinement against $|\vec{F}_{extrapolated}|$ or some variation thereof has become a standard method for the retrieval of the triggered state structure in time-resolved crystallography[6,7,46,77].

However, as stated above, the extrapolation process introduces errors, moreover, the occupancy of the triggered state needs to be known, and determining this value is nontrivial in protein crystallography, given the usually limited resolution of the diffraction data (see also the chapter below). In the present case, we used a method based on map values. In the system under study, the photolysis of carboxymyoglobin, a CO molecule is displaced from its binding position on the heme iron to a nearby binding pocket. Thus, in the dark state, the ground-state heme position is assumed to be fully occupied and have high electron density, whereas in a map showing exclusively the triggered (photolyzed) structure, the electron density at this position should be 0. Thus, to determine the unknown occupancy of the triggered state, we proceeded as follows: we calculated extrapolated structure factors assuming a range of triggered-state occupancies varying from 0.05 to 0.8, and monitored the electron density at the dark-state CO position. As can be seen above, $|\vec{F}_{extrapolated}|$ tends to $|\vec{F}_{dark}|$ as the assumed occupancy increases. Indeed, when the assumed occupancy becomes higher than the true occupancy, dark-

state features (such as a CO molecule in its dark-state position) start to become visible in the map. Thus, the map value at this position increases with increasing assumed occupancy, and as the correct, true occupancy we chose the occupancy where this map value crosses an admittedly arbitrary value of 1.0 σ, as this is where we would normally contour the map to look for dark-state features. We thus used this value as the true occupancy and used it to calculate extrapolated structure factors to determine the structure of the triggered state.

### 1.1.1 Benchmarking using Simulated Data

However, how successful is this method in retrieving the true triggered-state structure? To investigate this, we benchmarked our scripts using simulated data. To this end, we calculated datasets from partially photolyzed CO.myoglobin crystals by combining dark- and photolyzed structures into a single PDB file and calculating $\vec{F}_{light}$ from it using PHENIX. We also simulated dark-state data $\vec{F}_{dark}$. In both cases, a solvent model with $k_{sol} = 0.35$ and $B_{sol} = 50$ Å$^2$ was used, and 5% errors were added to the structure factors, as this resulted in datasets that were reasonably consistent with the peak heights in our experimental $|\vec{F}_{light}| - |\vec{F}_{dark}|$ maps. Given the error levels in our experimental structure factors ($R_{split} \approx 0.25$) we had naively expected to have to use 12.5% error levels given the square-root relationship between intensities and amplitudes, but this resulted in almost uninterpretable maps, which does not match our experimental observations.

We simulated data with triggered-state occupancies between 0.1 and 0.8 and subjected them to the same scripts for occupancy determination and structure factor extrapolation we have previously used for our experimental data[7]. After extrapolation, triggered-state structures were refined against the extrapolated structure factor amplitudes calculated from each of the simulated data sets. In each case, the dark-state structure was used as the starting structure for

the refinement, without any manual rebuilding other than placing the CO molecule in the triggered-state binding pocket (Supplemental Figure S3a).

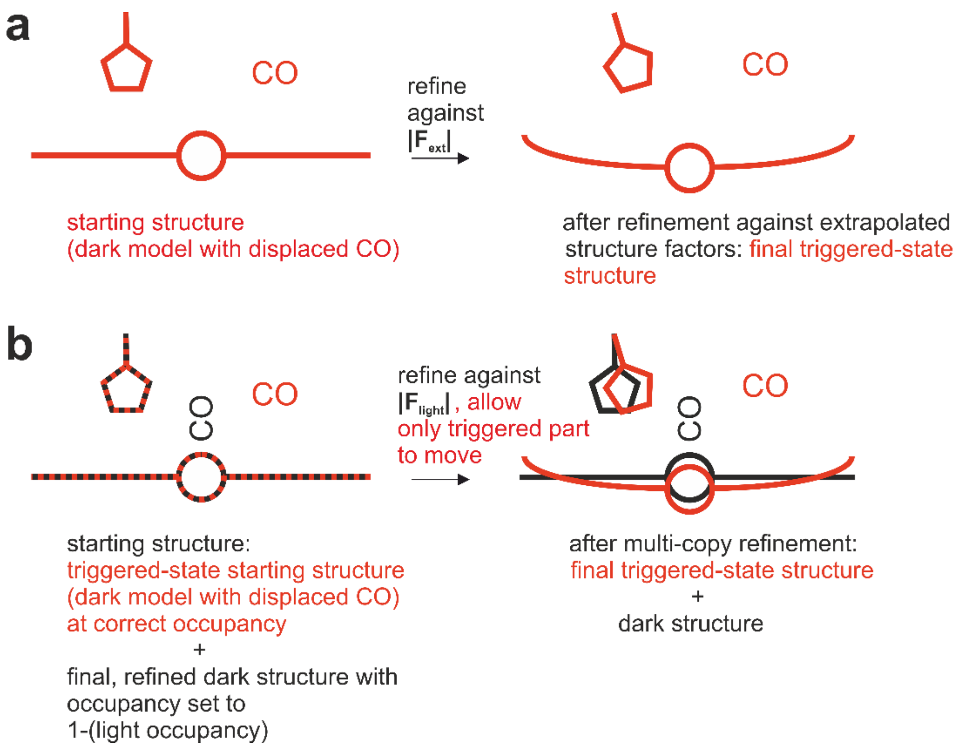


**Supplemental Figure S3. Refinement strategies**. a. Refinement against extrapolated structure factors involved only a triggered-state structure. b. Multi-copy refinement is done with a mixture of dark- and triggered-state structures at their respective occupancies, where only the triggered-state part is refined whereas the dark-state part remains constant.

We first tested our extrapolation-based method for occupancy retrieval, in which we calculate maps from extrapolated structure factors with increasing assumed occupancies, while monitoring the map value at the ground-state CO position. We matched the values obtained to the "true" occupancy, i.e., the triggered-state occupancy used in simulating the data sets. It was recently suggested[78] that the occupancies found in this way would be off by a factor of ½, because a map calculated from extrapolated structure factor amplitudes is equivalent to a dark-

state map to which a scaled difference map has been added, and difference map peaks are necessarily at half height. This is true in real space; however, we found that while this method indeed tends to underestimate the occupancies (Supplemental Figure S4a) it does not do so by a factor of ½ with these simulated data.

We next investigated how well the extrapolation-based methods retrieved the “true” triggered-state structure, i.e., the triggered-state structure used to simulate the data sets. As shown in (Supplemental Figure S4b, the triggered-state iron-out-of-plane distance used in the simulation (~0.29 Å) was reproduced reasonably well at higher occupancies, but clearly underestimated at occupancies of 0.3 and lower. Small conformational changes of individual residues are also reproduced reasonably well at high occupancies, but low occupancies may result in structures that are too close to the dark-state structure (Supplemental Figure S4c-h).

Thus, we conclude that while structure factor extrapolation can retrieve even small structural changes successfully, at low occupancies the method underestimates even a comparatively large structural change such as the displacement of an electron-rich iron atom. We therefore looked into other methods for triggered structure retrieval.

One such method would be the refinement of dark-and triggered-state structures together, at their respective occupancies, which is referred to as multi-copy refinement. Thus, one would combine the known dark-state structure at its occupancy with a starting model for the triggered state at its occupancy, and refine it against the observed $|\vec{F}_{light}|$. (Supplemental Figure S3b). By allowing only the triggered-state part to move one would greatly reduce the number of parameters. Moreover, in such a scheme, the refinement would actually simulate the crystal during the experiment and the structure factors calculated during refinement would correctly add the contributions of the dark- and triggered state models as complex numbers. In a sense, it is a true fitting of parameters to the actual experiment, whereas structure factor extrapolation is not. Thus, many of the assumptions made in structure factor approximation are not needed;

however, knowledge of the triggered-state occupancy would still be required, as in our experience, occupancy refinement with this type of data is not very stable.

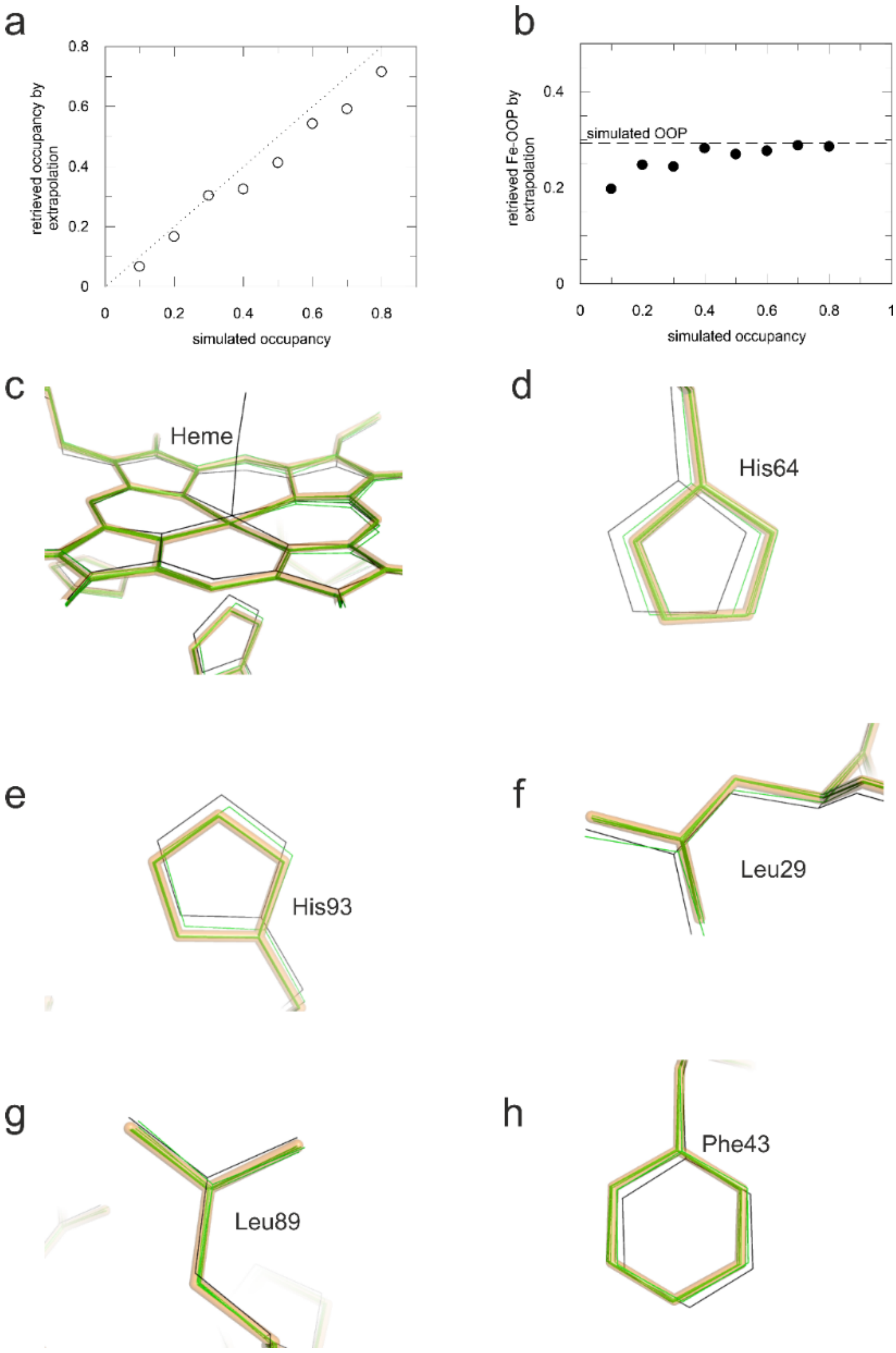
a
retrieved occupancy by extrapolation
simulated occupancy
0
0.2
0.4
0.6
0.8
b
retrieved Fe-OOP by extrapolation
simulated occupancy
simulated OOP
0
0.2
0.4
0.6
0.8
1
c
Heme
d
His64
e
His93
f
Leu29
g
Leu89
h
Phe43

**Supplemental Figure S4. a. Simulation results for the automated determination of occupancy from extrapolated data.** a. Data were simulated using predefined occupancies ranging from 0.1-0.8 (x-axis) and then subjected to automated occupancy determination. The result is plotted on the y-axis; the dashed line represents perfect retrieval of the true occupancy. b. Retrieved iron-out-of-plane distances from extrapolated data using simulated data; the true iron-out-of-plane distance is indicated by the dashed line. c-h. Success of the retrieval of triggered-state conformations of individual residues. The dark-state structure is shown as black lines, the triggered-state structure as transparent orange sticks, and the retrieved triggered structures for occupancies between 0.1 and 0.8 as green lines. In most cases the triggered-state structure is reproduced well; the outliers are largest at occupancy 0.1.

Importantly, we can again use our knowledge of the system to devise a scheme to determine the triggered-state occupancy using a map value at the dark-state CO position. If the assumed occupancy of the triggered state is incorrect, the occupancy of the dark state will be incorrect, too, and refinement of a model with such incorrect occupancies will result in $|\vec{F}_{obs}| - |\vec{F}_{calc}|$ difference electron density at the dark-state CO position. We thus did the following: against each of our simulated datasets we refined models with a range of triggered-state occupancies and checked the difference electron density map at the dark-state CO position, taking the occupancy where this value crosses 0 as the correct occupancy. We then used these occupancies to construct dark/triggered state mixtures and refined these against the respective data sets, allowing only the triggered state part of the model to move. Again, we used as the starting structure for the triggered state a dark-state model with a displaced CO but no other changes. Strikingly, with this multi-copy refinement method there was no systematic underestimation of the occupancy (Supplemental Figure S5a), and the iron-out-of-plane distance was

reproduced faithfully at all occupancies investigated (Supplemental Figure S5b). Moreover, small structural changes in individual residues were reproduced faithfully at all occupancies used (Supplemental Figure S5c-h). We conclude that for the system analyzed, the multi-copy method works better than the extrapolation method in terms of retrieving the true light-state structure, and therefore continued with this method.

A third method suggests itself (see also ref.[78]); as mentioned above, the calculation of a map from extrapolated structure factors with dark-state phases is equivalent to adding a scaled light-dark difference map to the dark-state map. Thus, any features introduced via the "difference map" part of the calculation are at ½ their true height. One can now determine these differences by subtracting the dark map from the extrapolated map in real space:

$$\rho_{diff} = \rho_{extrapolated} - \rho_{dark}$$

We could then scale these differences by a factor of 2 and add them back to the dark state map to calculate a corrected extrapolated map: $\rho_{corrected} = \rho_{dark} + 2\rho_{diff}$

Initial simulations suggest an improvement in map quality may be obtained, and experiments with back transformation of the corrected map to structure factor amplitudes for refinement have been initiated. It should be noted, however, that any noise introduced in the extrapolation process will also be increased by a factor of 2 in this method.

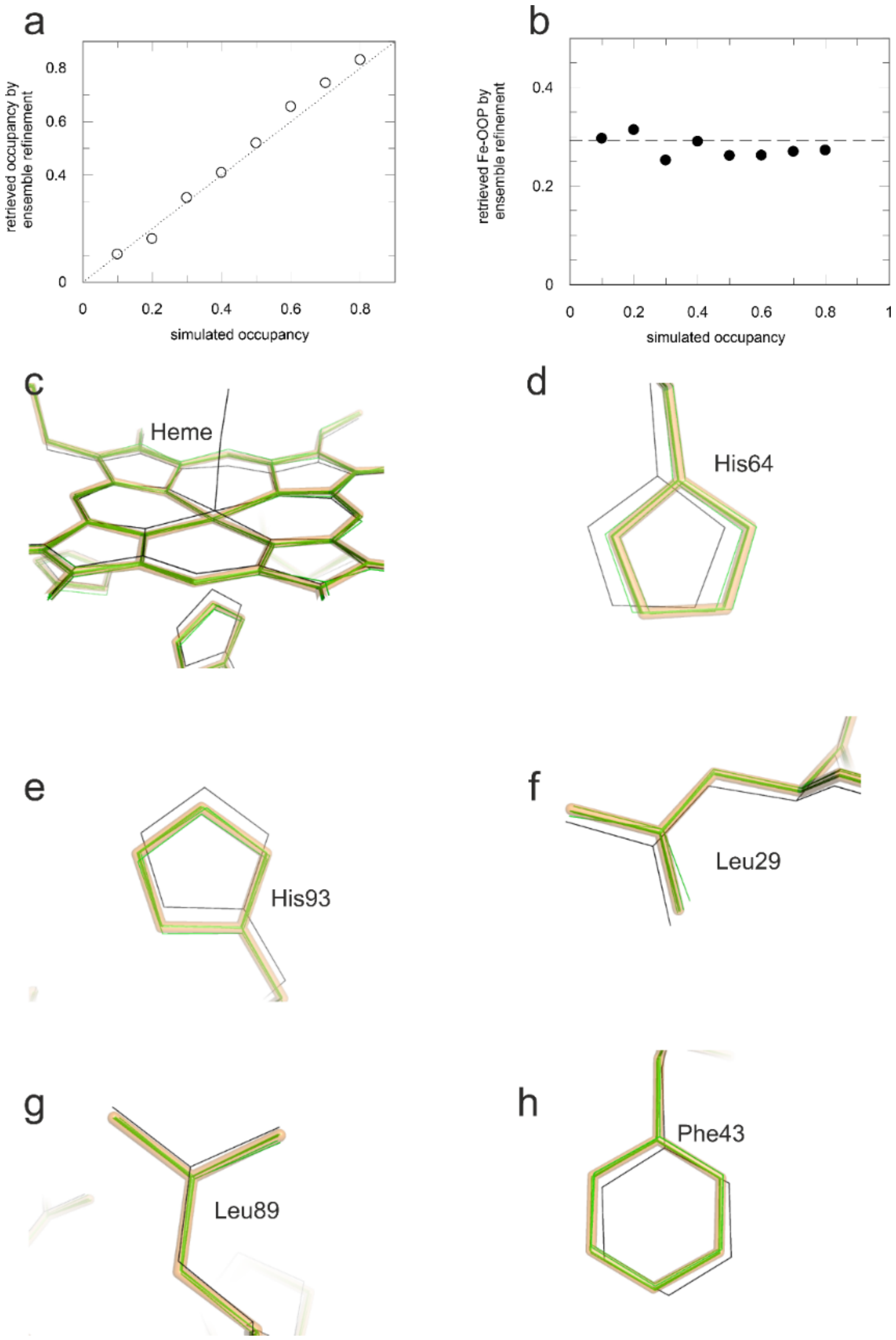

a
retrieved occupancy by ensemble refinement
simulated occupancy
0
0.2
0.4
0.6
0.8
b
retrieved Fe-OOP by ensemble refinement
simulated occupancy
1
c
Heme
d
His64
e
His93
f
Leu29
g
Leu89
h
Phe43

**Supplemental Figure S5. a. Simulation results for the automated determination of occupancy using multi-copy refinement.** a. Data were simulated using predefined occupancies ranging from 0.1-0.8 (x-axis) and then subjected to automated occupancy determination. The result is plotted on the y-axis; the dashed line represents perfect retrieval of the true occupancy. b. Retrieved iron-out-of-plane distances using multi-copy refinement against simulated data; the true iron-out-of-plane distance is indicated by the dashed line. c-h. Success of the retrieval of triggered-state conformations of individual residues. The dark-state structure is shown as black lines, the triggered-state structure as transparent orange sticks, and the retrieved triggered structures for occupancies between 0.1 and 0.8 as green lines.

### 1.2 Determination of photolyzed occupancy

Apart from the need to know the occupancy of the photolyzed state for the retrieval of the photolyzed structure from crystals with partial occupancies, the current paper bases some of its conclusions on the time evolution of the photolyzed occupancies. We therefore looked into various methods to determine this quantity in addition to those described in section 1.1 of this supplementary section.

First, in an effort to use as few assumptions as possible, we refined myoglobin models without any ligand (CO or otherwise) in either the ground-state CO site on the heme or the photolyzed-state binding pocket against our various datasets. For data from partially photolyzed MbCO crystals, this should result in an Fo-Fc difference electron density map with two peaks: one in the dark-state pocket close to the heme iron (denoted CO) and one in the photolyzed-state pocket (denoted CO*). Comparison of these peaks should give a measure of the occupancy of the photolyzed state. At first glance it would appear that the integrated value of these two peaks should be compared, but this poses the question of at which level to contour the maps so as to determine the extent of each peak. Moreover, we found that in our present data, despite the

high resolution, the two peaks often meld together even at relatively high sigma levels. We therefore compared the relative heights of the two peaks, using the formula

$$occupancy = \frac{\rho_{CO*}}{\rho_{CO} + \rho_{CO*}}$$

where $\rho_{CO*}$ and $\rho_{CO}$are the heights of the peaks for the photolyzed- and dark state, respectively. Applying this method to the power titration data results in the curves shown in Supplementary Figure S6a, which shows a smooth increase of the apparent photolysis yield with pump laser fluence, levelling off at high fluence. Importantly, the occupancies determined in this way closely mirror those found using the multi-copy refinement method described in section 1.1 of the Supplementary Note 1 (see main text Figure 1).

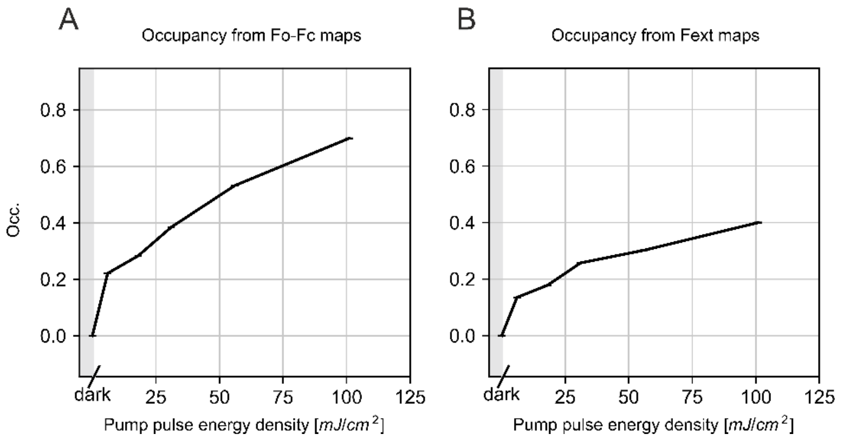


**Supplementary Figure S6**. **Occupancies of the photolyzed CO (CO*) from power titration data.** Determination **a.** from Fo-Fc difference electron density maps, **b**. from extrapolated maps Fext.

We then compared these results to those obtained using another method: inspection of electron density maps from extrapolated structure factors, as described in part I of this supplement. Briefly, we calculated extrapolated structure factors[62] using assumed occupancies increasing in small steps from 0.05 to 0.7. These were combined with dark-state model phases and electron

density maps were calculated. We then inspected these maps for the presence of dark-state features in the form of electron density for a dark-state CO (1 σ, as measured on the position of the CO oxygen atom), taking the occupancy at which this dark-state feature just becomes visible as the final value. This also resulted in smoothly increasing occupancies with increasing pump fluence, showing the same levelling off at high fluence, but with different absolute values (Supplemental Figure S6b). Indeed, the extrapolation method suggests lower values than the omit map method, which is consistent with the tendency to underestimate the occupancy noted in section 1.1 of this Supplementary Note 1.

Similar results were obtained with the time resolved data. We calculated occupancies for all time delays at all three pump laser fluences used, using Fo-Fc map peak heights as well as by looking for dark-state features in Fext maps. Moreover, using 100 datasets for each time point prepared by bootstrap resampling (see *coordinate error estimation*) we estimated errors for the resulting occupancy estimates. This resulted in the curves shown in Supplementary Figure S7. Again, the Fo-Fc omit map peak height method gave results that closely correspond to those found by multi-copy refinement. However, when comparing the omit map method to the extrapolation method, similar trends but different absolute values are seen, with extrapolation resulting in smaller values for the occupancy. To make matters worse, applying q-weighting[79] in the Fext-based procedure resulted in yet another set of occupancy values (not shown).

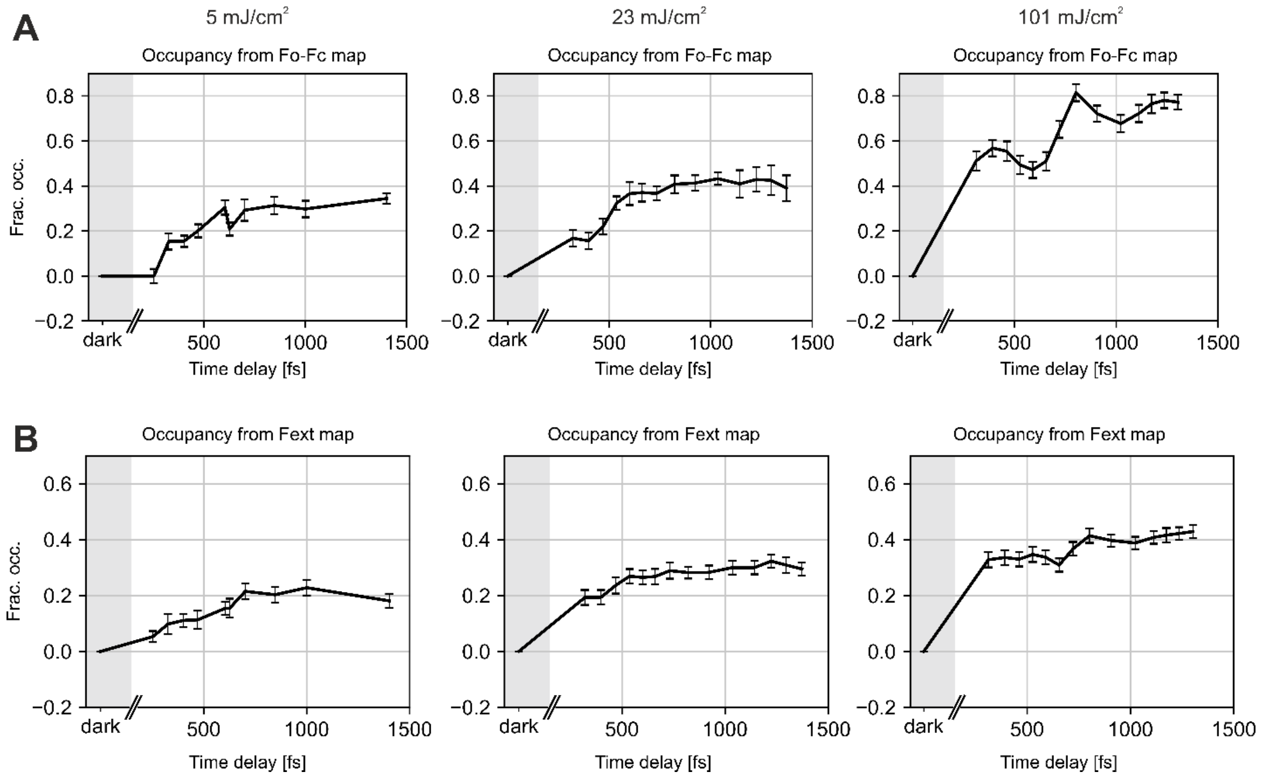


**Supplementary Figure S7**: **Occupancies for time-resolved data**. Determination from **A.** Fo-Fc difference electron density maps and **B**. Fext maps

We conclude that while the various methods result in different absolute values for the occupancies, with extrapolation possibly underestimating the true value, relative trends are conserved, and given the results of the bootstrapping, the precision of each of the methods is relatively high.

### 1.3 Feasibility of photolyzed-state structure retrieval from the 2.4 mJ/cm$^2$ data

To investigate whether useful structural information can be retrieved from the 2.4 mJ/cm$^2$ data, we focused on the 1.3 ps time delay of that data series, as this data set may be expected to have both the highest occupancy and the largest structural change upon photolysis, and therefore the strongest signal.

We therefore subjected these data to both the extrapolation- and multi-copy refinement schemes as described in section 1.1 of the Supplementary Note 1. In the extrapolation case (Figure S8a), an occupancy of 0.045 was obtained, but the structure did not show the expected features: the amount of heme doming was far less than expected for a photolyzed structure, the distal His64 showed only very little conformational change, the proximal His 93 showed a rotation around $\chi 1$ that is not typical for photolyzed carboxymyoglobin.

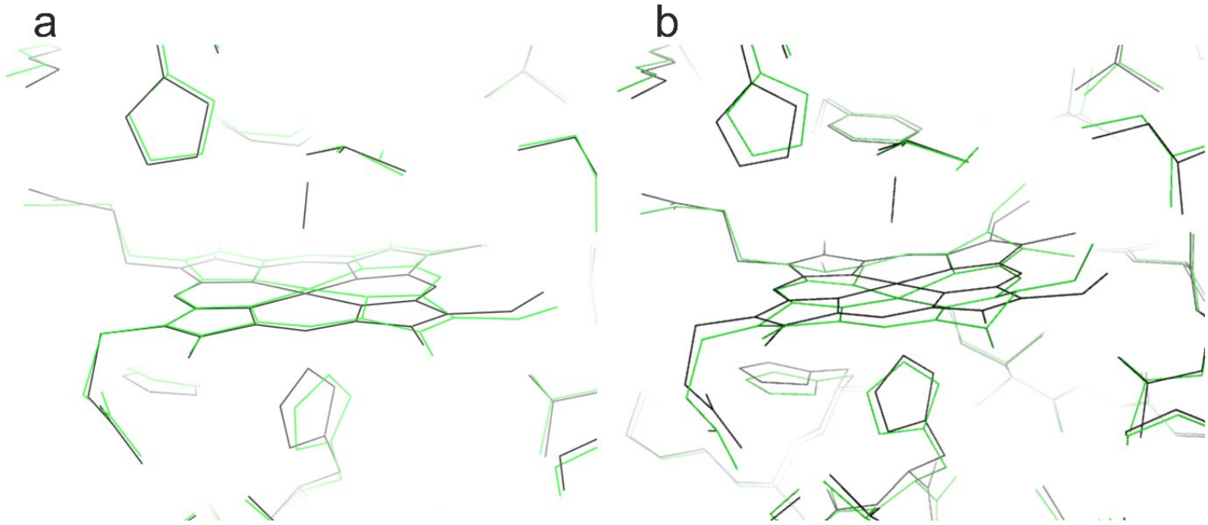


**Supplementary Figure S8. Results of photolyzed-state structure retrieval from the 1.3 ps time delay 2.4 mJ/cm$^2$ data**. **a.** refinement against extrapolated structure factors and **b.** multi-copy refinement. The dark-state structure is shown in black, the retrieved photolyzed structure in green.

Using the multi-copy refinement method, an occupancy of 0.03 was estimated. However, here, too, an unrealistic structure was obtained (Supplementary Figure S8b), in which the structure of the heme was severely distorted.

Thus, both methods (structure factor extrapolation and multi-copy refinement) apparently fail at retrieving a realistic structure from the photolyzed state from the 2.4 mJ/cm$^2$ data even at the longest time delay, which should have the highest occupancy and the largest structural state. However, both methods agree that the occupancy is on the order of a few percent for these data. This appears realistic, as there is only very little difference density in the light-dark map for this data set (Extended Data Figure 5).

Conceivably, at very low occupancy, the signal is too small for either method to be able to retrieve the photolyzed-state structure at the error levels present in these data. We therefore simulated mixtures with 5% light-state occupancy as described in section 1.1.1 of the Supplementary Note 1 but now at varying error levels, ranging from 5% to 40% of the structure factor amplitudes.

When these simulated data were subjected to automatic structure factor extrapolation, none of the retrieved structures faithfully reproduced the features of the photolyzed-state model used to simulate the data (Supplementary Figure S9a). Moreover, the retrieved occupancies were off by a factor of two in all cases – but in either direction, i.e., either upwards or downwards. When the multi-copy refinement method was used, similar observations were made using errors between 5 and 40% (Supplementary Figure S9b); the retrieved occupancies varied between 1 and 7 % and the retrieved structures were highly distorted and did not reproduce the "true" photolyzed-state structure used to simulate the data.

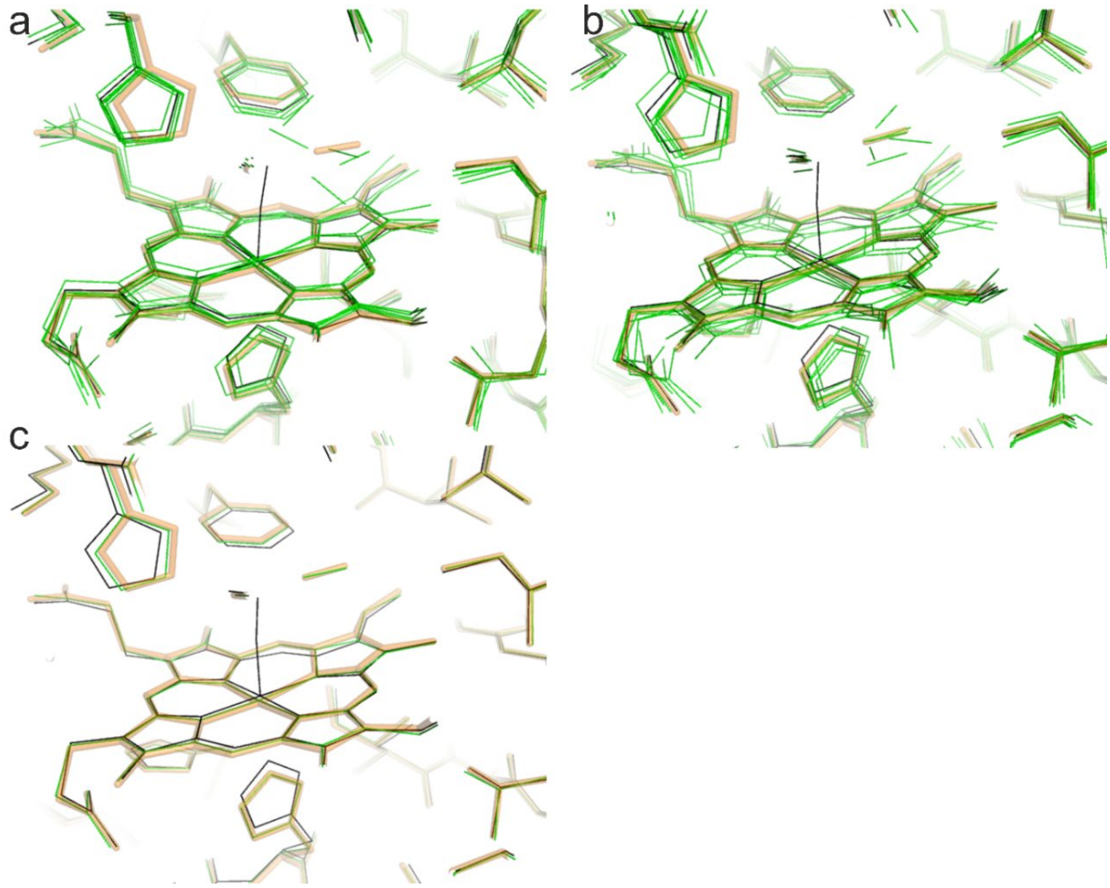


**Supplementary Figure S9. Photolyzed-state structure retrieval from the simulated data with an occupancy of 0.05 and error levels between 5 and 40%. a.** Refinement against extrapolated structure factors, b. multi-copy refinement, and **c**. as **b**, but only showing the 5% error result. The dark-state structure is shown in black, the retrieved photolyzed structures in green, and the "true" photolyzed-state structure as an orange outline.

As set out before (see section 1.1.1 of the Supplementary Note 1), these simulations appear to mirror the observed data described in the current manuscript best at simulated error levels of ~5%. Thus, the simulations are in line with the observations in the sense that the simulations also show that at the current error levels, the occupancy in the 2.4 mJ/cm$^2$ data is too low to allow retrieval of the photolyzed-state structure.

However, in serial crystallography, (stochastic) errors can be reduced by collecting more data; indeed, the signal-to-noise ratio should increase with the square root of the number of indexed

lattices (as illustrated in ref. [46]). We therefore investigated whether more data would have allowed us to retrieve the photolyzed-state structure from the 2.4 mJ/cm$^2$ data. To that end, we simulated data with an occupancy of 0.05 but with an error level of 2.5%. This corresponds to half the error levels in the simulations in in section 1.1 of the Supplementary Note 1, which in turn correspond to the estimated error levels in our observed data. Thus, these simulations assume half the error levels we estimate for the observed data, and therefore, collecting four times as many diffraction patterns would have allowed us to reach these error levels.

Strikingly, while the extrapolation method still failed at error levels of 2.5%, the multi-copy refinement method faithfully reproduced the photolyzed-state structure from simulated data at this lower error level (Supplementary Figure S9c). Thus, we tentatively conclude that the failure of the methods evaluated here (extrapolation and multi-copy refinement) to retrieve the photolyzed-state structure from the 2.4 mJ/cm$^2$ data is likely due to the error levels in the data. Moreover, our simulations suggest that this can be mitigated by increasing the number of indexed lattices, as the concomitant decrease in error levels may allow the multi-copy method at least to faithfully retrieve the structure of the photolyzed state. In general, 20,000 indexed lattices were used for each time-resolved data point. Collecting ~ 80,000 diffraction patterns/time point (to increase the signal two-fold) would be feasible.

It is noteworthy that the situation of our 2.4 mJ/cm$^2$ vs. 5 mJ/cm$^2$ data resembles the one reported for rhodopsin (Ext. Data Fig. 3c in ref. [14]) and photolyase[23], respectively. In both cases, two datasets were collected, using photoexcitation fluences differing by a factor of two (rhodopsin) or three (photolyase). Despite the observation of similar (although weaker) peaks in the $F^{light}_{obs\ (\Delta t)}$- $F^{dark}_{obs}$ difference electron density maps, the low fluence structures could not be refined using extrapolated structure factors. Our analysis suggests that it may be worthwhile revisiting the analysis of those data along the approaches outlined here. Moreover, collecting more data instead of increasing the laser energy should be considered to avoid controversy[19].

## 1.4 Coordinate Error Estimation

As the present manuscript considers small changes in interatomic distances, a careful analysis of coordinate errors needs to be made. In small-molecule crystallography, where the diffraction data typically extend to well beyond 1 Å resolution, full-matrix refinement can be performed which yields error estimates for every parameter refined. In protein crystallography, such resolution is typically not attained and consequently, full-matrix refinement is not possible, precluding estimation of errors for each parameter. Instead, statistical methods such as those proposed by Cruickshank[80] or methods based on maximum likelihood[81] are used to estimate average coordinate errors, and therefore lump together parameters that are likely very well determined, such as the positions of heavy atoms, with less well determined parameters, such as the positions of atoms at the distal ends of flexible side chains. Importantly, these methods were all devised for rotation crystallography.

In the present case, serial crystallography was used for data collection, and to our knowledge, no formalism for the estimation of average coordinate errors has been devised for such data yet.

Moreover, most of the structures discussed in the current manuscript are based on multi-copy refinement, and it is not clear how this process affects the estimation of an overall error for that part of the structure that is of interest (i.e., the photolyzed part). However, serial crystallography does lend itself naturally to resampling methods such as bootstrapping[46], which was therefore used in this case.

Moreover, bootstrapping (and comparable resampling methods) do allow errors for individual parameters to be estimated. We will compare the results for both multi-copy refinement and extrapolation, using the 327 fs time delay structure at 5 mJ/cm$^2$ pump pulse fluence. Intuitively, one would expect large coordinate errors for this structure, given that it has a low occupancy, which should result in large errors in the retrieved structures.

We therefore analyzed the bootstrapping results of the 5 mJ/cm$^2$ 327 fs structure. The $R_{free}$ for this structure is indeed 21.8%. Applying the formula proposed by Murshudov and Dodson[82] for the estimation of average coordinate error one obtains an estimate of 0.07 Å for the average coordinate error in this structure. This overall coordinate error would appear to be at odds with the individual error bars shown in *e.g.* Extended Data Figure 7. However, it is an overall coordinate error, and does not consider local variances in coordinate precision.

$$\langle \sigma_x^2 \rangle \approx 0.65 \cdot \frac{n_a}{n_o} \cdot R_{free}^2 \cdot d_{min}^2 \cdot C^{3/2}$$

(formula suggested by Murshudov and Dodson[82] for the overall error in a structure as $\sqrt{\langle \sigma_x^2 \rangle}$, where $n_a$ is the number of atoms, $n_o$ the number of observed structure factor amplitudes, $R_{free}$ is the free R-factor, $d_{min}$ the resolution and $C$ the completeness.)

Indeed, estimating the coordinate error $\sigma(x)$ for each atom individually from 100 structures obtained by bootstrap resampling (averaging the errors along the *x*, *y* and *z* axes for each atom) results in a very broad distribution of errors, ranging from 0.01 Å to 0.24 Å, with an average of 0.04 Å. This average coordinate error, while smaller than that estimated from $R_{free}$, is much larger than several of the error bars shown in Extended Data Figure 7. However, this analysis also shows that the coordinates of some atoms are much more well defined than others. Mapping these errors onto the structure reveals that, not surprisingly, atoms belonging to- or in close proximity to the main chain of the protein have smaller coordinate errors than those further away; for the distal His64, for instance, $\sigma(x)$ for the main-chain C atom is only 0.02 Å, whereas for the Cε1 and Nε2 atoms, $\sigma(x)$ is >0.05 Å.

Importantly, a particularly well-defined atom in terms of $\sigma(x)$ in the structure is, as expected, the heme iron atom, with an average coordinate error from bootstrapping of 0.018 Å. In the

CO-dissociated state, this atom is bound by five other atoms, restricting its mobility, and moreover, it is the heaviest atom in the structure in terms of the electron density, meaning that its position can be more readily determined using X-ray crystallography. Thus, one would expect relatively small error bars compared to the average error for those quantities depending on the iron position, such as the iron-out-of-plane distance and the heme-His93 N distance. Other well-defined atoms are the heme pyrrole N atoms, and the Cα atoms of the protein. As most of the quantities discussed in the current paper depend on the coordinates of these atoms, one may expect error bars for them that are considerably smaller than one would expect from the average, "overall" coordinate error estimates derived from quantities such as $R_{free}$ or even the smaller average coordinate error obtained from bootstrapping.

We conclude that bootstrapping suggests that the structures display overall stochastic errors that are on the order of what might be expected from the formula suggested by Murshudov and Johnson, but that these are indeed overall errors, the magnitude of which depends on the local structure.

## Supplementary Note 2

TR-SFX pump probe experiments face conflicting demands with respect to crystal dimensions: high-resolution diffraction typically benefits from thick crystals whereas efficient photoexcitation requires optically thin samples. Specifically, absorption as described by Lambert-Beer's law sets a strict upper limit for light penetration of a given wavelength, absorption coefficient and chromophore concentration. Due to the high chromophore concentration in crystalline samples (typically 10-30 mM), this in turn translates into a need for very small crystal dimensions. One can change the pump wavelength to reduce absorption and thereby allow use of larger crystals, but only while bearing in mind any possible concomitant changes in cross sections of the various pathways. These often reduce the yield

of the desired photoproduct[83,84] which, after all, is ultimately the quantity to be optimized. Thus, experimental conditions need to be identified such that maximal photoconversion is obtained in the probed crystal volume upon excitation with a femtosecond laser pulse, while yet ensuring that the photoproduct yield is intrinsically limited only by internal conversion reactions[83].

In pump probe TR-SFX experiments a quick estimate of the photoproduct yield – or that of an ensuing reaction intermediate – can be obtained by calculating $F_{obs}^{light}$- $F_{obs}^{dark}$ difference electron density maps and plotting the height of the positive and negative peaks (corresponding to appearance and disappearance of light and dark state, respectively) as a function of laser energy (see Fig. 1a,b). Typically, if the signal seems inadequate, the laser energy is then increased. Another pump probe SFX dataset is collected and new difference electron density maps calculated. The objective, however, must not be to simply *increase* peak heights until usable signal results. Valid characterization of biologically relevant single photon-induced reactions can only be made in the regime of *linearly increasing* signal with pump laser energy. Establishing the linear regime requires experimental power titrations. Nonlinearities observed at high pump fluences can arise due to various factors (absorption saturation leading to side reactions triggered upon multiphoton absorption such as stimulated emission, photoionization, …). Consequently, power density is critical, meaning that both energy density and pulse length of the pump laser play a role.

If the crystal size exceeds the 1/e penetration depth of the pump laser wavelength, high laser fluence often seems unavoidable in obtaining adequate photoexcitation. This is because very few photons reach the "rear" regions of the crystals, forming a "pedestal" of dark molecules within each crystal (Extended Data Fig. 2). The resulting small apparent $F_{obs}^{light}$- $F_{obs}^{dark}$ difference is simply due to inadequate excitation of chromophores in this pedestal at low laser

fluence. Note also that it is impossible to adequately excite these “rear” molecules with a single laser without also overexposing the “front” molecules, resulting in their multiphoton excitation. Often it is assumed that this is not an issue for thin plate-shaped crystals. For randomly oriented crystals (as in SFX) this is incorrect.

A frequent justification for using relatively large crystals for time-resolved experiments is that smaller crystals often do not diffract to high resolution, in particular crystals of very anisotropic dimensions (needles, plates). However, this issue can be addressed by fracturing larger well-ordered crystals into microcrystalline parts[48].

There are several reasons that the light-induced signal can be much lower than expected: (i) losses due to scattering, for example at air-liquid interfaces, resulting in lower than expected laser fluence at the crystal, (ii) unfavorable orientation of the absorption dipole moment of the chromophore **μ** and the electric field vector **E**. The probability of excitation is proportional to $(\boldsymbol{\mu} \cdot \mathbf{E})^2$ and thus $\sim\cos^2\theta$, with θ being the angle of the laser polarization vector and the chromophore transition dipole. This effect is particularly noticeable at low laser fluence, and in crystals significantly more so than in solution. Since the orientations of chromophores within a crystal are anything but stochastic, it can even be that for certain crystal orientations no photons at all are absorbed. (iii) stimulated emission (SE). Whether a photon is absorbed or whether it stimulates emission depends on the ground and excited state populations (the Einstein coefficients for absorption and SE are the same). Loss through SE dominates at high laser fluence, see for example reference [85].

For jet-based measurements, scattering losses through (i) are typically ≤20 % for non-scattering carrier media such as LCP and other transparent media. However, any optically small scattering centers (small water droplets in grease[8] or other tiny particles in the crystal-

surrounding medium, notably the particles intrinsic to Super Lube® [86]) result in strong isotropic scattering of the pump light within the jet. This makes quantification of the effective laser irradiance at the crystal extremely difficult. Crystals outside of the direct light path can be illuminated, as can crystals upstream of the illumination zone[87]. Simple transmission measurements then yield misleading effective fluences[8]. A general problem when reporting scattering losses at jet interfaces is the neglect of the strong lens effect of the cylindrical surface of the jet[26], resulting in erroneously high reported losses of ~80 %[6].

## Supplementary Note 3: Quantum chemistry

The initial steps of the MbCO photolysis are now well established[21]. Absorption of a photon to the singlet Q state (Supplementary Figure S10a) is followed by transfer to the singlet metal-to-ligand charge-transfer (MLCT) band due to a strong Jahn-Teller distortion in the excited state. This affords an energy transfer from the porphyrin plane to the Fe-center, activating dissociative Fe-CO stretching vibrations and thus CO dissociation (see Supplementary Figure S10b). Sequential Fe spin transitions ensue, ultimately resulting in the formation of a high-spin FeII center.

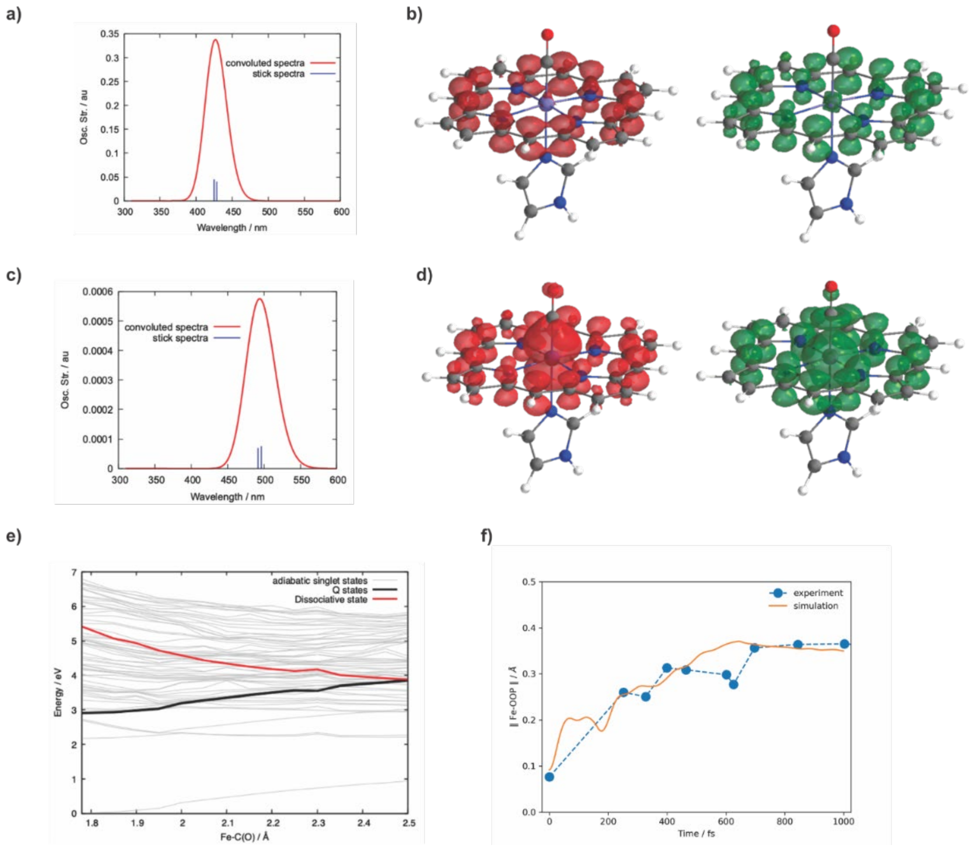

**Supplementary Figure S10**: **Quantum chemical analysis of photon absorption to the Q state of carboxymyoglobin. a**) Single photon absorption. Absorption spectrum of the heme-CO model ground state; **b**) Attachment-detachment density analysis of the most intense transition in this spectral region, corresponding to the Q states of porphyrin. In red, the electron density is depleted and in green the electron density is increased. **c**) Sequential two-photon absorption. Absorption spectrum of the Q-band excited heme-CO model. **d**) Attachment-detachment density analysis of the most intense transition in this spectral region, corresponding to a dissociative Fe-CO state. In red, the electron density is depleted and in green the electron density is increased. **e**) Potential energy curve of 60 electronic singlet states along a relaxed scan on the Fe-C(O) dissociation. The grey thin lines represent the adiabatic states. A diabatic character is represented in thicker colored lines; the black thick lines represent the Q state and the red thick line depicts the dissociative state. **f**) cumulative running average (orange line) of the Fe-OOP coordinate as a function of time for the 40 QM/MM trajectories overlaid with the single photon excitation experimental data (5 mJ/cm$^2$) (blue points).

We showed recently that the Jahn-Teller distortion ultimately induces coherent oscillations of the Fe-CO bond distance[21]. The predicted damping time is in line with the apparent increase of the occupancy of photolyzed CO with time (Fig. 2a)), interpreted as decreasing "disorder" of the position of the dissociated CO with time. However, with increasing photon flux, the apparent heme-CO photolysis appears "accelerated" and, eventually, the apparent occupancy of CO hardly changes with time (Fig. 2a, ref. [3]). We explain this observation by sequential two-photon absorption. The first photon leads to the Q-band excitation of heme-CO (Supplementary Figure S10a) corresponding to a $\pi \rightarrow \pi^*$ transition in porphyrin. This state can absorb a second

photon. The absorption spectrum of the Q-excited heme-CO system is shown in Supplementary Figure S10c, in which a high-energy singlet state is populated, corresponding to roughly two times the energy of the Q state. Analysis of its excitation character shows that this state is mixed π→π* of the heme and $d_{xy}$→$d_{z2}$/ $d_{yz}$→$d_{z2}$ character with respect to the ground state, and therefore is dissociative for the Fe-CO bond (see Supplementary Figure S10d).

We performed a relaxed scan of the potential-energy surface (PES) cut of the singlet manifold along the Fe-C(O) dissociation coordinates (see Methods, Computational Details section). Supplementary Fig. 10e depicts the lowest 60 electronic states in grey. The black lines represent the Q states, the red line represents the higher-energy dissociative state (Supplementary Figure S10e). It is clear that upon excitation to the dissociative singlet, the excited wave packet experiences a large gradient towards Fe-C(O) dissociation and due to the (barrierless) repulsive nature of the potential, no coherent oscillation of the wave packet is expected on this state.

The Fe out-of-plane (OOP) motion is one of the quantities that is measurable from the experimental data during the MbCO photolysis. The dynamics of myoglobin following photodissociation of CO has been simulated at the QM/MM level. It can be assumed that the difference in the energies of the bright states and the lowest $^5$MLCT state is deposited as kinetic energy to the atoms closest to the chromophore. The amount of excess energy (0.1 eV) is redistributed randomly to the kinetic energy of the QM atoms of the simulation box. From this, 40 initial conditions were generated and propagated up to 1 ps at the DFT/BHHLYP/6-31G* level. The ensemble average of the Fe-OOP coordinate was computed as a function of time. The results of the simulation agree well with the experimentally derived ones (see Supplementary Figure S10f). The Fe-OOP coordinate starts from its equilibrium value (approximately 0.1 Ang.) in MbCO and increases with time up to shortly after 600 fs. The Fe-

OOP coordinate does not change significantly after 600 fs both in experimental and simulation results.

In order to interpret the dynamics at different time delays, we analyzed the protein cavity changes during a relaxed scan coordinate from a sudden dissociation of CO (Supplementary Figure S11). The reaction coordinate (Supplementary Figure S11a) corresponds to the different Fe-CO distances.

The scans show conformational changes in both the heme (Supplementary Figure S11b-e) and the surrounding protein (Supplementary Figure S11f-j) along this reaction coordinate, which in several cases show the same trends as observed by time-resolved SFX (Extended Data Fig. 7 and 9), such as for the heme CHD-Ile99 CD1 distance, the Phe43 $\chi 2$ torsion angle and the iron out-of-plane motion.

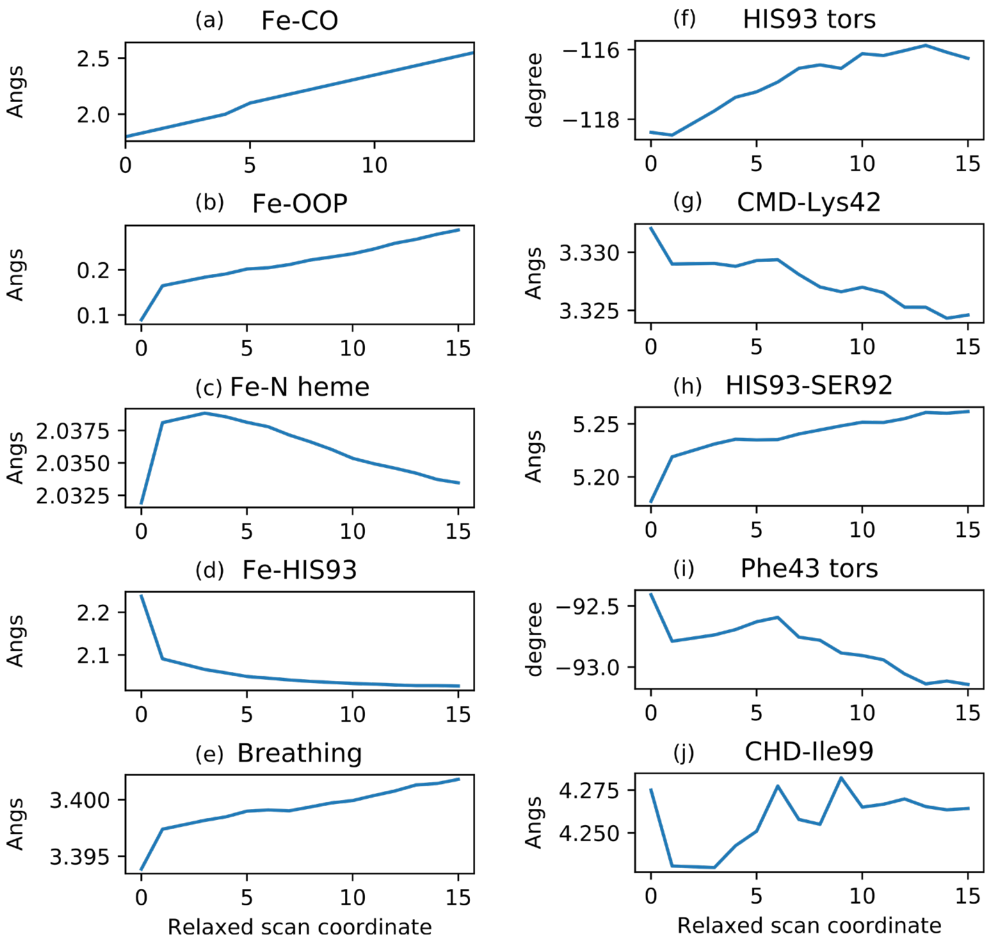


**Supplementary Figure S11. Changes in the heme and protein cavity motions along a relaxed scan coordinate in the protein model. a**) definition of the relaxed scan coordinate at fixed Fe-CO distances. **b**) Iron out-of-plane (FeOOP) motion. **c**) Average iron-nitrogen (Fe-Np) distance. **d**) Iron-nitrogen distance from the proximal histidine His93 (Fe-$N_{His93}$). **e**) Heme in-plane breathing mode. **f**) His93 $\chi2$ torsion angle, **g**) distance between the heme CMD atom and the Lys32 backbone carbonyl atom, **h**) length of the His93 ND1…Ser92OG hydrogen bond, **i**) Phe43 $\chi2$ torsion angle, **j**) heme CHD-Ile99-CD1 distance. See also Extended Data Fig. 8.

**Supplementary Figures cited in the main text**

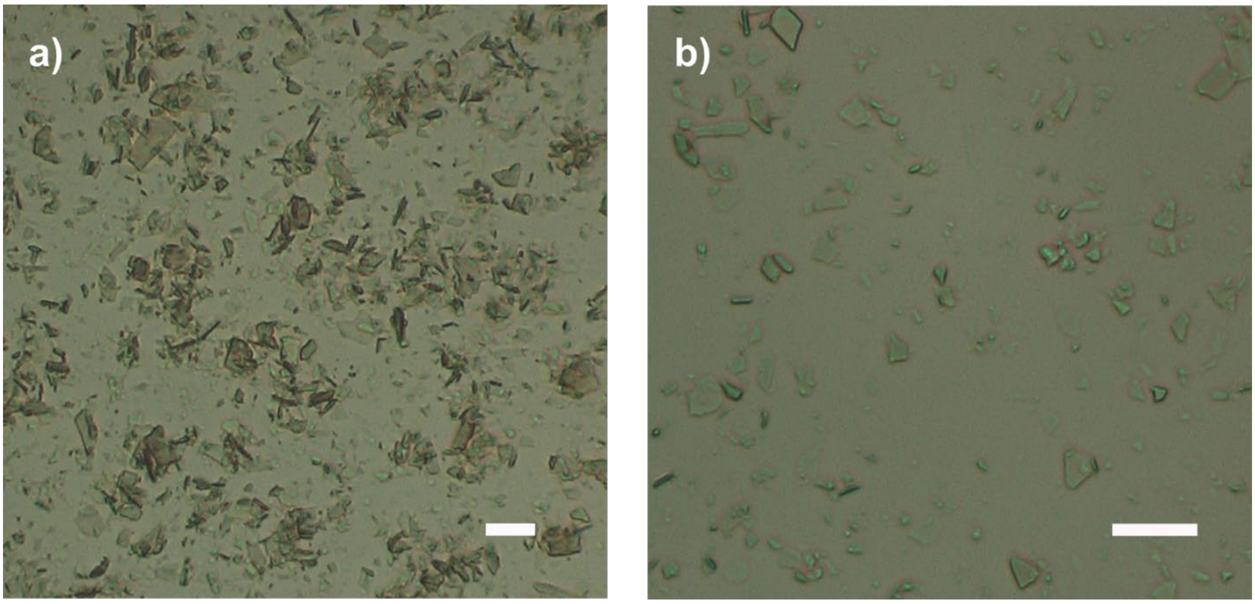


**Supplementary Figure S12**

Macroscopic myoglobin crystals were fractured by filtration using tandem array stainless steel ¼ inch diameter filters[48]. For the first beamtime two filter stacks were used consisting of 100, 40 µm filters followed by a second tandem array of 40, 20, 10, 10, 5 µm. This resulted in the crystallites shown in (a), with many of the larger crystals showing lengths of ~ 15-20 µm. For the second beamtime the crystalline slurry was further fractionated using a tandem array of 10, 5, 2, 2 µm filters. This resulted in the crystallites shown in (b). The larger crystals are less than 10 µm long. In both images the white scale bar corresponds to 20 µm. Images were taken with a Hirox microscope.

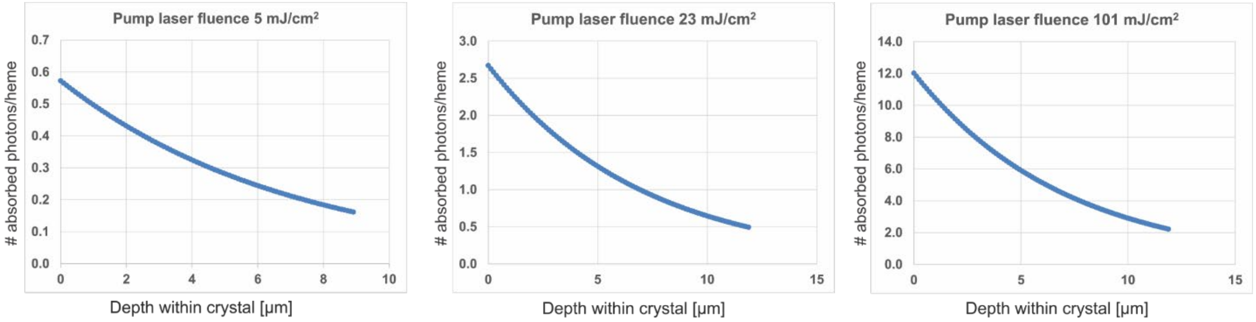


**Supplementary Figure S13. Average number of nominally absorbed photons.** The number of nominally absorbed photons/heme in photoexcited monoclinic myoglobin crystals was calculated for different pump laser energies as described recently[26]. It was assumed that the first and subsequently absorbed photons have the same absorption cross sections. As indicated (see Extended Data Table 2), the crystals used for SFX data collection of the 5 mJ/cm$^2$ laser fluence data were smaller than those used for the higher fluence photoexcitation data.

## Supplementary Tables

Supplementary Table 1a – Data collection and model statistics, power titration

| **Data set**<br>**pdb entry** | **dark**<br>**8bkh** | **6 mJ/cm$^2$**<br>**8r8f** | **18 mJ/cm$^2$**<br>**8r8g** | **31 mJ/cm$^2$**<br>**8r8h** | **56 mJ/cm$^2$**<br>**8r8i** | **101 mJ/cm$^2$**<br>**8r8j** |
|---|---|---|---|---|---|---|
| Space group | | | --- $P2_1$ --- | | | |
| unit cell | | | | | | |
| *a,b,c* [Å] | | | --- 64.1 28.8 35.8 --- | | | |
| α, β, γ [°] | | | --- 90.0 106.9 90.0 --- | | | |
| Resolution [Å] | 30.67-1.35 (1.39-1.35) | 10.00-1.33 (1.37-1.33) | 10.00-1.33 (1.37-1.33) | 10.00-1.33 (1.37-1.33) | 10.00-1.39 (1.43-1.39) | 10.00-1.33 (1.37-1.33) |
| No. indexed lattices[1] | 19,193 | 19258 | 18465 | 28225 | 12767 | 15911 |
| I/σ(I) | 4.0 (1.2) | 4.2 (1.2) | 4.1 (1.2) | 4.8 (1.4) | 3.6 (1.3) | 4.0 (1.2) |
| $R_{split\,[\%]}$ | 22.4 (86.0) | 21.3 (83.2) | 20.9 (83.5) | 18.0 (73.7) | 26.8 (78.9) | 21.5 (88.1) |
| CC* | 0.982 (0.72) | 0.983 (0.745) | 0.985 (0.702) | 0.988 (0.783) | 0.971 (0.692) | 0.984 (0.709) |
| Completeness [%] | 100.0 (99.6) | 100.0 (99.5) | 99.9 (99.2) | 100.0 (99.9) | 100.0 (99.8) | 99.9 (99.1) |
| Multiplicity | 121.4 (33.1) | 125.7 (30.4) | 119.4 (29.0) | 181.9 (44.2) | 87.2 (30.3) | 105.3 (25.5) |
| Wilson B [Å$^2$] | 15.2 | 15.1 | 15.3 | 15.3 | 15.8 | 15.6 |
| Res. used [Å] | 20.44-1.35 | 10.0-1.4 | 10.0-1.4 | 10.0-1.4 | 10.0-1.4 | 10.0-1.4 |
| Occupancy | n.a. | 0.16 | 0.24 | 0.37 | 0.54 | 0.74 |
| $R/R_{free}$ | 0.1833/ 0.2271 | 0.1796 /0.2168 | 0.1788/ 0.2148 | 0.1755/ 0.2131 | 0.1891/ 0.2299 | 0.1813/ 0.2174 |
| No. atoms | | | | | | |
| Protein | 1194 | 2388 | 2388 | 2388 | 2388 | 2388 |
| Ligand/ion | 55 | 110 | 110 | 110 | 110 | 110 |
| Water | 112 | 224 | 224 | 224 | 224 | 224 |
| *B*-factors | | | | | | |
| Protein | 18.4 | 18.0 | 18.1 | 18.0 | 18.4 | 18.3 |
| Ligand/ion | 16.0 | 16.1 | 16.4 | 16.3 | 16.7 | 16.6 |
| Water | 28.6 | 27.7 | 28.0 | 28.0 | 28.4 | 28.3 |
| RMS bond deviations | | | | | | |
| lengths [Å] | 0.007 | 0.010 | 0.009 | 0.009 | 0.009 | 0.009 |
| angles [°] | 1.00 | 1.336 | 1.133 | 1.133 | 1.090 | 1.047 |

[1] As many images as practicable during the available beam time were collected, and all indexed lattices available for a certain pump fluence were included in each data set, causing the number of lattices to differ between data sets

## Supplementary Table 1b – Data collection and model statistics, 5 μJ/cm$^2$ time series

| Data set<br>pdb entry | dark<br>8bkn | 254 fs (nom. 150)<br>8r8w | 327 fs (nom. 225)<br>8r8x | 402 fs (nom. 300)<br>8r8y | 471 fs (nom. 375)<br>8r8z | 604 fs (nom. 450)<br>8r90 | 627 fs (nom. 525)<br>8r91 | 702 fs (nom. 600)<br>8r92 | 847 fs (nom. 750)<br>8r93 | 1001 fs (nom. 900)<br>8r94 | 1401 fs (nom. 1300)<br>8r95 |
|---|---|---|---|---|---|---|---|---|---|---|---|
| Space group | $P2_1$ | | | | | ----- $P2_1$ ----- | | | | | |
| unit cell | | | | | | ----- 63.1, 28.4, 35.3 ----- | | | | | |
| $a,b,c$ [Å] | | | | | | ----- 90, 107, 90 ----- | | | | | |
| α, β, γ [°] | | | | | | | | | | | |
| Resolution [Å] | 30.12-1.29 (1.32-1.29) | 30.17-1.32 (1.35-1.32) | 30.17-1.35 (1.39-1.35) | 30.17-1.32 (1.35-1.32) | 30.17-1.32 (1.35-1.32) | 30.17-1.27 (1.30-1.27) | 30.17-1.35 (1.39-1.35) | 30.17-1.32 (1.35-1.32) | 30.17-1.32 (1.35-1.32) | 30.17-1.32 (1.35-1.32) | 30.17-1.27 (1.30-1.27) |
| No. indexed lattices[1] | 63,521 | 29,916 | 9,667 | 27,781 | 14,418 | 33,341 | 13,767 | 27,396 | 24,926 | 25,888 | 23,896 |
| I/σ(I) | 5.7 (1.6) | 4.2 (1.4) | 3.0 (1.0) | 4.2 (1.3) | 3.2 (1.1) | 4.1 (1.1) | 3.2 (1.2) | 4.1 (1.5) | 4.0 (1.4) | 4.1 (1.4) | 3.8 (0.9) |
| $R_{split\,[\%]}$ | 14.7 (65.8) | 21.2 (75.5) | 34.0 (90.6) | 21.4 (78.5) | 29.6 (97.2) | 20.4 (112.4) | 32.0 (83.7) | 22.3 (77.6) | 22.4 (77.2) | 22.2 (75.7) | 23.3 (105.4) |
| CC* | 0.992 (0.835) | 0.982 (0.815) | 0.954 (0.654) | 0.983 (0.764) | 0.968 (0.693) | 0.986 (0.718) | 0.959 (0.747) | 0.982 (0.783) | 0.981 (0.801) | 0.982 (0.804) | 0.979 (0.745) |
| Completeness [%] | 99.9 (99.1) | 99.9 (98.6) | 99.5 (94.9) | 99.9 (98.7) | 99.4 (94.4) | 99.6 (96.5) | 99.7 (97.4) | 99.8 (98.3) | 99.8 (97.8) | 99.9 (98.7) | 99.2 (93.5) |
| Multiplicity | 264.9 (42.9) | 129.7 (28.5) | 47.5 (13.8) | 124.9 (27.5) | 62.6 (14.2) | 132.7 (17.5) | 62.4 (17.3) | 121.8 (27.2) | 111.8 (24.5) | 119.9 (26.5) | 103.5 (13.9) |
| Wilson B [Å$^2$] | 13.7 | 14.1 | 13.9 | 14.1 | 13.9 | 13.8 | 14.0 | 13.9 | 13.8 | 14.1 | 13.5 |
| Res. used [Å] | 30.12-1.29 | 10.0-1.4 | 10.0-1.4 | 10.0-1.4 | 10.0-1.4 | 10.0-1.4 | 10.0-1.4 | 10.0-1.4 | 10.0-1.4 | 10.0-1.4 | 10.0-1.4 |
| Occupancy | n.a. | 0.36 | 0.36 | 0.36 | 0.36 | 0.36 | 0.36 | 0.36 | 0.36 | 0.36 | 0.36 |
| $R/R_{free}$ | 0.1750/ 0.2095 | 0.1735/ 0.2073 | 0.1942/ 0.2181 | 0.1766/ 0.2049 | 0.1871/ 0.2169 | 0.1746/ 0.2056 | 0.1907/ 0.2249 | 0.1750/ 0.2055 | 0.1752/ 0.2001 | 0.1755/ 0.2082 | 0.1764/ 0.2074 |
| No. atoms | | | | | | | | | | | |
| Protein | 1194 | 2388 | 2388 | 2388 | 2388 | 2388 | 2388 | 2388 | 2388 | 2388 | 2388 |
| Ligand/ion | 55 | 110 | 110 | 110 | 110 | 110 | 110 | 110 | 110 | 110 | 110 |
| Water | 112 | 260 | 260 | 260 | 260 | 260 | 260 | 260 | 260 | 260 | 260 |
| *B*-factors | | | | | | | | | | | |
| Protein | 16.7 | 16.8 | 16.8 | 16.8 | 16.9 | 17.1 | 16.7 | 16.7 | 16.7 | 16.8 | 16.7 |
| Ligand/ion | 14.0 | 14.2 | 14.1 | 14.2 | 14.3 | 14.4 | 14.1 | 14.1 | 14.1 | 14.2 | 14.1 |
| Water | 27.7 | 28.1 | 28.1 | 28.1 | 28.2 | 28.4 | 28.1 | 28.0 | 28.0 | 28.1 | 28.1 |
| RMS bond deviations | | | | | | | | | | | |
| lengths [Å] | 0.010 | 0.010 | 0.010 | 0.011 | 0.011 | 0.010 | 0.011 | 0.011 | 0.012 | 0.011 | 0.011 |
| angles [°] | 1.03 | 1.117 | 1.082 | 1.154 | 1.146 | 1.093 | 1.211 | 0.188 | 1.232 | 1.174 | 1.216 |

[1] As many images as practicable during the available beam time were collected, and all indexed lattices available for a certain pump fluence were included in each data set, causing the number of lattices to differ between data sets

# Supplementary Table 1c – Data collection and model statistics, 23 µJ/cm$^2$ time series

| Data set<br>pdb entry | dark*<br>8bkh | 320 fs<br>8r9c | 399 fs<br>8r9d | 469 fs<br>8r9e | 537 fs<br>8r9f | 600 fs<br>8r9g | 661 fs<br>8r9h | 733 fs<br>8r9i | 822 fs<br>8r9j | 922 fs<br>8r9k | 1036 fs<br>8r9l | 1143 fs<br>8r9m | 1224 fs<br>8r9n | 1297 fs<br>8r9p | 1373 fs<br>8r9q |
|---|---|---|---|---|---|---|---|---|---|---|---|---|---|---|---|
| Space group | $P2_1$ | | | | | ----- $P2_1$ ----- | | | | | | | | | |
| unit cell | | | | | | | | | | | | | | | |
| *a,b,c* [Å] | 64.1 28.8 35.8 | | | | | ----- 64.2, 28.9, 35.9 ----- | | | | | | | | | |
| α, β, γ [°] | 90.0 106.9 90.0 | | | | | ----- 90, 107.0, 90 ----- | | | | | | | | | |
| Resolution [Å] | 30.67-1.35 (1.39-1.35) | 30.70-1.32 (1.35-1.32) | 30.70-1.32 (1.35-1.32) | 30.70-1.32 (1.35-1.32 | 30.70-1.32 (1.35-1.32) | 30.70-1.32 (1.35-1.32) | 30.70-1.32 (1.35-1.32) | 30.70-1.35 (1.39-1.35) | 30.70-1.35 (1.39-1.35) | 30.67-1.32 (1.35-1.32) | 30.67-1.32 (1.35-1.32) | 30.65-1.32 (1.35-1.32) | 30.70-1.32 (1.35-1.32) | 30.70-1.32 (1.35-1.32) | 30.70-1.32 (1.35-1.32) |
| No. indexed lattices[1] | 19,193 | 20,000 | 20,000 | 20,000 | 20,000 | 20,000 | 20,000 | 20,000 | 20,000 | 20,000 | 20,000 | 20,000 | 20,000 | 20,000 | 20,000 |
| I/σ(I) | 4.0 (1.2) | 3.7 (1.2) | 3.7 (1.1) | 3.7 (1.2) | 3.6 (1.2) | 3.6 (1.5) | 3.5 (1.1) | 3.7 (1.3) | 3.9 (1.3) | 4.0 (1.1) | 4.1 (1.1) | 3.8 (1.1) | 3.7 (1.3) | 3.6 (1.2) | 3.6 (1.6) |
| $R_{split [\%]}$ | 22.4 (86.0) | 23.1 (86.8) | 22.9 (88.1) | 23.5 (89.2) | 24.2 (90.9) | 24.9 (97.2) | 25.0 (94.9) | 23.8 (80.4) | 23.2 (78.7) | 21.9 (91.6) | 21.1 (92.0) | 22.4 (92.3) | 23.9 (89.5) | 24.6 (91.1) | 24.6 (93.5) |
| CC* | 0.982 (0.72) | 0.981 (0.764) | 0.981 (0.767) | 0.979 (0.709) | 0.98 (0.695) | 0.978 (0.657) | 0.977 (0.723) | 0.979 (0.771) | 0.98 (0.807) | 0.982 (0.71) | 0.983 (0.616) | 0.982 (0.624) | 0.979 (0.677) | 0.977 (0.626) | 0.978 (0.667) |
| Completeness [%] | 100.0 (99.6) | 99.8 (97.2) | 99.8 (97.3) | 99.8 (97.3) | 99.8 (97.2) | 99.8 (97.7) | 99.8 (97.6) | 99.9 (99.3) | 100.0 (99.7) | 99.9 (99.2) | 99.9 (99.3) | 99.8 (98.2) | 99.8 (97.6) | 99.7 (96.9) | 99.8 (97.3) |
| Multiplicity | 121.4 (33.1) | 82.3 (17.8) | 83.1 (17.9) | 83.6 (18.1) | 82.7 (17.9) | 81.6 (17.6) | 80.5 (17.4) | 89.4 (24.0) | 103.9 (27.8) | 117.3 (25.5) | 119.6 (26.3) | 101.2 (22.0) | 87.2 (18.9) | 82.3 (18.0) | 81.4 (17.5) |
| Wilson B [Å$^2$] | 15.2 | 14.4 | 14.4 | 14.3 | 14.4 | 14.4 | 14.4 | 14.8 | 15.0 | 15.0 | 15.1 | 14.9 | 14.4 | 14.3 | 14.4 |
| Res. used [Å] | 20.44-1.35 | 10.0-1.4 | 10.0-1.4 | 10.0-1.4 | 10.0-1.4 | 10.0-1.4 | 10.0-1.4 | 10.0-1.4 | 10.0-1.4 | 10.0-1.4 | 10.0-1.4 | 10.0-1.4 | 10.0-1.4 | 10.0-1.4 | 10.0-1.4 |
| Occupancy | n.a. | 0.479 | 0.479 | 0.479 | 0.479 | 0.479 | 0.479 | 0.479 | 0.479 | 0.479 | 0.479 | 0.479 | 0.479 | 0.479 | 0.479 |
| $R/R_{free}$ | 0.1833/ 0.2271 | 0.1843/ 0.2201 | 0.1833/ 0.2180 | 0.1832/ 0.2204 | 0.1842/ 0.2201 | 0.1849/ 0.2222 | 0.1849/ 0.2216 | 0.1840/ 0.2245 | 0.1825/ 0.2262 | 0.1810/ 0.2237 | 0.1815/ 0.2223 | 0.1833/ 0.2242 | 0.1837/ 0.2249 | 0.1845/ 0.2267 | 0.1853/ 0.2243 |
| No. atoms | | | | | | | | | | | | | | | |
| Protein | 1194 | 2388 | 2388 | 2388 | 2388 | 2388 | 2388 | 2388 | 2388 | 2388 | 2388 | 2388 | 2388 | 2388 | 2388 |
| Ligand/ion | 55 | 110 | 110 | 110 | 110 | 110 | 110 | 110 | 110 | 110 | 110 | 110 | 110 | 110 | 110 |
| Water | 112 | 224 | 224 | 224 | 224 | 224 | 224 | 224 | 224 | 224 | 224 | 224 | 224 | 224 | 224 |
| *B*-factors | | | | | | | | | | | | | | | |
| Protein | 18.4 | 17.2 | 16.9 | 17.1 | 17.2 | 17.3 | 17.3 | 17.5 | 17.6 | 18.0 | 18.0 | 17.9 | 17.8 | 17.2 | 17.2 |
| Ligand/ion | 16.0 | 15.5 | 15.2 | 15.4 | 15.5 | 15.6 | 15.6 | 15.8 | 15.9 | 16.3 | 16.3 | 16.2 | 16.1 | 15.5 | 15.5 |
| Water | 28.6 | 27.2 | 26.9 | 27.1 | 27.2 | 27.3 | 27.2 | 27.4 | 27.6 | 28.0 | 28.0 | 27.9 | 27.7 | 27.2 | 27.2 |
| RMS bond deviations | | | | | | | | | | | | | | | |
| lengths [Å] | 0.007 | 0.009 | 0.009 | 0.009 | 0.009 | 0.010 | 0.009 | 0.009 | 0.009 | 0.009 | 0.009 | 0.009 | 0.010 | 0.010 | 0.009 |
| angles [°] | 1.00 | 1.039 | 1.056 | 1.053 | 1.078 | 1.182 | 1.106 | 1.072 | 1.079 | 1.061 | 1.079 | 1.070 | 1.146 | 1.165 | 1.090 |

* the dark state data are the same as those used for the power titration- and 101 mJ/cm$^2$ fluence experiments

[1] The 23 and 101 mJ/cm$^2$ fluence time delay data were collected at several nominal time delay settings, indexed and combined into single set of indexed lattices. These lattives were then sorted by their actual time delay as determined by the timing tool and binned into individual data sets of 20,000 lattices each (with an overlap of 10,000 lattices on either side). See Materials and Methods for details.

## Supplementary Table 1d – Data collection and model statistics, 101 µJ/cm$^2$ time series

| **Data set** / **pdb entry** | **dark*** **8bkh** | **312 fs** **8ra1** | **392 fs** **8ra2** | **462 fs** **8ra3** | **527 fs** **8ra4** | **590 fs** **8ra5** | **655 fs** **8ra6** | **722 fs** **8ra7** |
|---|---|---|---|---|---|---|---|---|
| Space group | *P*2$_1$ | | | | | ----- *P*2$_1$ ----- | | |
| unit cell | | | | | | | | |
| *a,b,c* [Å] | 64.1 28.8 35.8 | | | | | ----- 64.2, 28.9, 35.9 ----- | | |
| α, β, γ [°] | 90.0 106.9 90.0 | | | | | ----- 90, 107.0, 90 ----- | | |
| Resolution [Å] | 30.67-1.35 (1.39-1.35) | 30.65-1.32 (1.35-1.32) | 30.65-1.32 (1.35-1.32) | 30.70-1.32 (1.35-1.32) | 30.70-1.35 (1.39-1.35) | 30.70-1.35 (1.39-1.35) | 30.70-1.32 (1.35-1.32) | 30.70-1.35 (1.39-1.35) |
| No. indexed lattices[1] | 19,193 | 20,000 | 20,000 | 20,000 | 20,000 | 20,000 | 20,000 | 20,000 |
| I/σ(I) | 4.0 (1.2) | 3.5 (1.1) | 3.6 (1.2) | 3.5 (1.1) | 3.6 (1.3) | 3.7 (1.3) | 3.5 (1.0) | 3.7 (1.3) |
| $R_{split [\%]}$ | 22.4 (86.0) | 25.3 (96.8) | 24.2 (95.5) | 24.3 (97.3) | 24.6 (81.6) | 24.3 (81.5) | 24.0 (105.8) | 23.8 (79.6) |
| CC* | 0.982 (0.72) | 0.976 (0.63) | 0.98 (0.641) | 0.981 (0.636) | 0.978 (0.779) | 0.979 (0.728) | 0.98 (0.676) | 0.98 (0.779) |
| Completeness [%] | 100.0 (99.6) | 99.8 (98.4) | 99.8 (98.0) | 99.8 (98.3) | 100.0 (99.6) | 99.9 (99.4) | 99.8 (97.8) | 99.9 (99.1) |
| Multiplicity | 121.4 (33.1) | 78.4 (16.9) | 79.2 (17.1) | 79.0 (17.0) | 82.4 (22.1) | 83.5 (22.3) | 80.3 (17.4) | 86.6 (23.4) |
| Wilson B [Å$^2$] | 15.2 | 14.4 | 14.5 | 14.6 | 14.7 | 14.7 | 14.5 | 14.8 |
| Res. used [Å] | 20.44-1.35 | 10.0-1.4 | 10.0-1.4 | 10.0-1.4 | 10.0-1.4 | 10.0-1.4 | 10.0-1.4 | 10.0-1.4 |
| Occupancy | n.a. | 0.78 | 0.78 | 0.78 | 0.78 | 0.78 | 0.78 | 0.78 |
| $R/R_{free}$ | 0.1833/ 0.2271 | 0.1854/ 0.2218 | 0.1856/ 0.2211 | 0.1862/ 0.2168 | 0.1861/ 0.2184 | 0.1844/ 0.2193 | 0.1845/ 0.2180 | 0.1854/ 0.2234 |
| No. atoms | | | | | | | | |
| Protein | 1194 | 2388 | 2388 | 2388 | 2388 | 2388 | 2388 | 2388 |
| Ligand/ion | 55 | 110 | 110 | 110 | 110 | 110 | 110 | 110 |
| Water | 112 | 224 | 224 | 224 | 224 | 224 | 224 | 224 |
| *B*-factors | | | | | | | | |
| Protein | 18.4 | 18.2 | 18.3 | 18.3 | 18.5 | 18.5 | 18.3 | 18.4 |
| Ligand/ion | 16.0 | 16.6 | 16.6 | 16.7 | 16.8 | 16.8 | 16.7 | 16.7 |
| Water | 28.6 | 28.2 | 28.6 | 28.3 | 28.5 | 28.5 | 28.3 | 28.4 |
| RMS bond deviations | | | | | | | | |
| lengths [Å] | 0.007 | 0.009 | 0.009 | 0.009 | 0.009 | 0.009 | 0.009 | 0.009 |
| angles [°] | 1.00 | 1.018 | 1.008 | 1.013 | 1.018 | 1.002 | 1.030 | 1.054 |

| **Data set** / **pdb entry** | **802 fs** **8ra8** | **907 fs** **8ra9** | **1023 fs** **8raa** | **1112 fs** **8rab** | **1174 fs** **8rac** | **1235 fs** **8rad** | **1303 fs** **8rae** |
|---|---|---|---|---|---|---|---|
| Space group | | | | | | | |
| unit cell | | | | | | | |
| *a,b,c* [Å] | | | | | | | |
| α, β, γ [°] | | | | | | | |
| Resolution [Å] | 30.70-1.32 (1.35-1.32) | 30.65-1.35 (1.39-1.35) | 30.65-1.35 (1.39-1.35) | 30.65-1.32 (1.35-1.32) | 30.65-1.32 (1.35-1.32) | 30.70-1.35 (1.39-1.35) | 30.70-1.35 (1.39-1.35) |
| No. indexed lattices[1] | 20,000 | 20,000 | 20,000 | 20,000 | 20,000 | 20,000 | 20,000 |
| I/σ(I) | 3.7 (1.6) | 4.1 (1.4) | 4.2 (1.3) | 3.9 (1.2) | 3.7 (1.1) | 3.8 (1.2) | 3.8 (1.4) |
| $R_{split [\%]}$ | 23.4 (90.8) | 22.2 (77.1) | 21.1 (77.4) | 21.9 (98.7) | 23.5 (95.3) | 24.2 (77.2) | 23.8 (77.6) |
| CC* | 0.981 (0.735) | 0.982 (0.795) | 0.984 (0.791) | 0.983 (0.657) | 0.98 (0.765) | 0.978 (0.666) | 0.98 (0.685) |
| Completeness [%] | 99.8 (97.8) | 99.9 (99.2) | 100.0 (99.6) | 99.9 (98.2) | 99.8 (98.4) | 99.9 (99.2) | 99.9 (98.7) |
| Multiplicity | 87.8 (19.1) | 110.4 (30.2) | 121.3 (33.5) | 104.7 (22.7) | 94.3 (20.5) | 90.4 (24.1) | 86.1 (23.0) |
| Wilson B [Å$^2$] | 14.7 | 15.2 | 15.4 | 15.3 | 14.9 | 14.8 | 14.8 |
| Res. used [Å] | 10.0-1.4 | 10.0-1.4 | 10.0-1.4 | 10.0-1.4 | 10.0-1.4 | 10.0-1.4 | 10.0-1.4 |
| Occupancy | 0.78 | 0.78 | 0.78 | 0.78 | 0.78 | 0.78 | 0.78 |
| $R/R_{free}$ | 0.1850/ 0.2245 | 0.1830/ 0.2172 | 0.1822/ 0.2146 | 0.1835/ 0.2165 | 0.1840/ 0.2192 | 0.1861/ 0.2195 | 0.1871/ 0.2247 |
| No. atoms | | | | | | | |
| Protein | 2388 | 2388 | 2388 | 2388 | 2388 | 2388 | 2388 |
| Ligand/ion | 110 | 110 | 110 | 110 | 110 | 110 | 110 |
| Water | 224 | 224 | 224 | 224 | 224 | 224 | 224 |
| *B*-factors | | | | | | | |
| Protein | 18.4 | 18.6 | 18.8 | 18.7 | 18.5 | 18.4 | 18.4 |
| Ligand/ion | 16.7 | 17.0 | 17.2 | 17.1 | 16.8 | 16.7 | 16.8 |
| Water | 28.4 | 28.6 | 28.8 | 28.8 | 28.5 | 28.4 | 28.4 |
| RMS bond deviations | | | | | | | |
| lengths [Å] | 0.009 | 0.009 | 0.009 | 0.009 | 0.009 | 0.009 | 0.009 |
| angles [°] | 1.033 | 1.023 | 1.034 | 1.035 | 1.048 | 1.022 | 1.052 |

* the dark state data are the same as those used for the power titration- and 25 mJ/cm$^2$ fluence experiments

[1] The 23 and 101 mJ/cm$^2$ fluence time delay data were collected at several nominal time delay settings, indexed and combined into single set of indexed lattices. These lattices were then sorted by their actual time delay as determined by the timing tool and binned into individual data sets of indexed lattices each (with an overlap of 10,000 lattices on either side). See Materials and Methods for details.

Supplementary Table 2

| | Pulse energy (µJ) | Laser fluence ($mJ/cm^2$)[%] | Laser power ($GW/cm^2$)[%] | largest crystal dimension ⊥ jet flow[¶] (µm) | Average # absorbed photons* | # absorbed photons front side of crystal[¥] | # absorbed* photons back side of crystal[¥] |
|---|---|---|---|---|---|---|---|
| 1st beamtime[#] | 1[¿] | 6 | 75 | ~12 | 0.3 | 0.7 | 0.1 |
| | 3.1[¿] | 18 | 233 | ~12 | 1.0 | 2.0 | 0.4 |
| | 4 | 23 | 301 | ~12 | 1.3 | 2.7 | 0.5 |
| | 5[¿] | 31 | 376 | ~12 | 1.6 | 3.3 | 0.6 |
| | 10[¿] | 56 | 763 | ~12 | 3.2 | 6.6 | 1.2 |
| | 18 | 101 | 1355 | ~12 | 5.8 | 12 | 2.2 |
| 2nd beamtime[$] | 0.5[Ø] | 2.4 | 40 | ~9 | 0.2 | 0.3 | 0.08 |
| | 1.0 | 4.8 | 81 | ~9 | 0.3 | 0.6 | 0.2 |
| Barends 2015[3] | 5 | 57 | 380 | ~5 | 4.8[☹] | 6.7 | 3.3 |

[%] Maximum intensity in center of the Gaussian beam. It is not only the energy density (fluence) that matters but also the power density (irradiance). The latter takes into account the pulse duration. Very high power can lead to very high electric field strengths that can result in strong polarization effects or multi-photon ionization. The linear regime and the specific side reactions depend on the properties of the chromophore.

# Pump laser parameters: 70-80 fs (calculated), 530 ± 9 nm FWHM, spot size (120 µm (hor) x 130 µm (ver) FWHM), circularly polarized

¿ Power titration data only, no time-series collected (only for rows shown with grey background)

Ø At short time-delays the light-induced signal was too weak for crystallographic analysis

$ Pump laser parameters: 70-80 fs (calculated, 60 fs ± 5 fs measured), 530 ± 9 nm FWHM, spot size (150 µm (hor) x 120 µm (ver) FWHM)

¶ The crystals flow-align in the liquid microjet, with the longest crystal dimension oriented along the jet axis. The 1/e penetration depth of ~530 nm light is ~7 µm for the monoclinic MbCO crystals

*Assuming equal cross sections for the first and subsequent absorption events

¥ See Extended Data Fig. 2, Supplementary Fig. S13

☹Publication[3] states "~ one photon/heme ". This is not correct.

76. Drenth, J. Principles or Protein X-ray Crystallography, (Springer New York, NY, 1999).
77. De Zitter, E., Coquelle, N., Oeser, P., Barends, T.R.M. & Colletier, J.P. Xtrapol8 enables automatic elucidation of low-occupancy intermediate-states in crystallographic studies. Commun. Biol. 5, 640 (2022).
78. Schmidt, M. Practical considerations for the analysis of time-resolved x-ray data. Struct. Dyn. 10, 044303 (2023).
79. Ursby, T. & Bourgeois, D. Improved estimation of structure-factor difference amplitudes from poorly accurate data. Acta Crystallogr. A 53, 564-575 (1997).
80. Cruickshank, D.W.J. Remarks about protein structure precision. Acta Cryst. D 55, 583-601 (1999).
81. Murshudov, G.N., Vagin, A.A. & Dodson, E.J. Refinement of macromolecular structures by the maximum-likelihood method. Acta Cryst. D 53, 240-55 (1997).
82. Murshudov, G.N. & Dodson, E.J. Simplified error estimation a la Cruickshank in macromolecular crystallography. CCP4 Newsletter 33, https://legacy.ccp4.ac.uk/newsletters/newsletter33/murshudov.html (1997).
83. Lincoln, C.N., Fitzpatrick, A.E. & van Thor, J.J. Photoisomerisation quantum yield and non-linear cross-sections with femtosecond excitation of the photoactive yellow protein. Phys. Chem. Chem. Phys. 14, 15752-15764 (2012).
84. Kim, J.E., Tauber, M.J. & Mathies, R.A. Wavelength dependent cis-trans isomerization in vision. Biochem. 40, 13774-13778 (2001).
85. Groot, M.L., vanGrondelle, R., Leegwater, J.A. & vanMourik, F. Radical pair quantum yield in reaction centers of photosystem II of green plants and of the bacterium Rhodobacter sphaeroides. Saturation behavior with sub-picosecond pulses. J. Phys. Chem. B 101, 7869-7873 (1997).
86. Sugahara, M. et al. Grease matrix as a versatile carrier of proteins for serial crystallography. Nat. Methods 12, 61-3 (2015).
87. Li, H. et al. Capturing structural changes of the S1 to S2 transition of photosystem II using time-resolved serial femtosecond crystallography. IUCrJ 8, 431-443 (2021).
